# Relationship between Posttraumatic Stress Disorder and Sleep Disturbances in Syrian Refugees in the United States

**DOI:** 10.1055/s-0043-1768646

**Published:** 2023-06-07

**Authors:** Safa Sankari, Nancy Wrobel, Michelle Leonard, Lana Grasser, Abdulghani Sankari, Arash Javanbakht

**Affiliations:** 1 College of Arts, Sciences, and Letters, Behavioral Sciences, University of Michigan-Dearborn, Dearborn, Michigan, United States; 2 Department of Psychiatry and Behavioral Neurosciences, Wayne State University School of Medicine, Detroit, Michigan, United States; 3 Division of Pulmonary, Critical Care and Sleep Medicine, Department of Internal Medicine, Wayne State University School of Medicine, Detroit, Michigan, United States

**Keywords:** sleep, PTSD, trauma, refugee health, refugees

## Abstract

**Background** Posttraumatic stress disorder (PTSD) is associated with disturbed sleep. However, the impact of sleep disturbances and PTSD symptomology in refugee populations is not well known. This study examined how PTSD-related sleep symptoms and overall sleep quality were impacted by previous and current traumatic and stressful experiences.

**Methods** Adult Syrian refugees living in Southeast Michigan were assessed via scheduled in-home interviews. Overall sleep quality was measured using the Pittsburgh Sleep Quality Index. PTSD-related sleep disturbances were measured using the Pittsburgh Sleep Quality Index Addendum. The presence of PTSD symptomatology was assessed via self-report using the Posttraumatic Stress Disorder Checklist. The Life Events Checklist for the Diagnostic and Statistical Manual of Mental Disorders, Fifth Edition-5 screened for prior traumatic events experienced and the Postmigration Living Difficulties Questionnaire was assessed for postmigration stressors. Correlational analysis was conducted between overall sleep quality, PTSD symptom severity, and previous trauma experienced. A stepwise linear regression analysis was conducted to examine the role of overall sleep quality, PTSD-specific sleep disturbances, current living difficulties, and the number of preimmigration traumatic events directly experienced or witnessed due to the presence of overall PTSD symptomology.

**Results** A total of 53 adults completed the study. PTSD-disturbed sleep was found to be positively associated with overall poor sleep quality ( *r* = 0.42, *p* < 0.01), PTSD symptomology ( *r* = 0.65, *p* < 0.01), and current living difficulties ( *r* = 0.37, *p* < 0.05). The PTSD-related sleep disturbances (B = 0.66, *p* < 0.01) and postmigration living difficulties (B = 0.44, *p* < 0.01) were found to be the strongest predictors of PTSD symptoms.

**Conclusion** Disturbed sleep is strongly associated with current stressful experiences and PTSD symptomology among Syrian refugees.

## Introduction

The Syrian crisis that began in 2011 has brought about the massive displacement of Syrians both inside and outside of the country with humanitarian consequences unparalleled in modern times. 1 2 According to the United Nations High Commissioner for Refugees, over 13 million Syrians have been displaced since 2011, including 5.6 million refugees seeking refuge mostly in neighboring countries and more than 6.9 million remain internally displaced in Syria. 3

Sleep disturbances are the most common symptoms refugees report experiencing, which likely impair their functioning and may worsen or predicate other disorders. 4 For example, 92% of Abkhaz refugees reported insomnia, a type of sleep disturbance, and attributed the onset of their sleep difficulties to war and migratory trauma and stress. 5 Such sleep disturbances persisted up to 15 years postdisplacement. 5

While the literature shows a link between sleep disturbances and posttraumatic stress, 6 less is known about sleep disturbances in refugee populations. Other studies have also suggested that patients with posttraumatic stress disorder (PTSD) experience specific sleep disturbances such as trauma-related nightmares, night-time intrusive memories not related to the trauma experienced, hot flashes, general nervousness, and episodes of acting out dreams such as kicking, punching, running, or screaming which contribute to overall poor sleep quality. 7 8 9 10 11

**Fig. 1:**
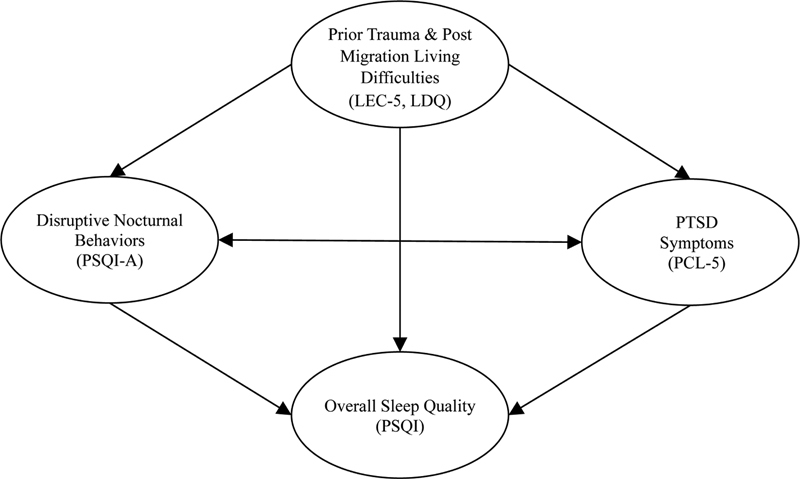
Proposed model of relationship between war related trauma and sleep and PTSD measures among sample of Syrian refugees. Legend: LDQ: Postmigration Living Difficulties Questionnaire, LEC: Life Events Checklist, PSQI: Pittsburgh Sleep Quality Index, PSQI-A: Pittsburgh Sleep Quality Index Addendum, PCL-5: Posttraumatic Checklist.

Therefore, the aim of the present study was to examine the relationship between overall sleep quality and specific posttraumatic stress-related sleep disturbances, otherwise known as disruptive nocturnal behaviors (DNB), in adult Syrian refugees. DNB following traumatic experiences consists of a constellation of nocturnal symptoms including abnormal behavior or movements or vocalization during sleep associated with autonomic hyperarousal. 12 Importantly, while most studies have queried facets of refugee health in border countries, no study has assessed refugees' sleep quality resettled in the United States. This is important as it examines the unique effects of living difficulties related to resettling in a host country of greater distance, cultural, and geographic distinction from the country of origin. These factors may confer a higher prevalence of postmigration living difficulties. We hypothesized DNB would be positively correlated with postmigration living difficulties and past trauma experienced by Syrian refugees. We also hypothesized that DNB would explain a significant amount of variance in posttraumatic stress above and beyond that of postmigration living difficulties and prior trauma ( Fig. 1 ).

## Methods

### Participants

Data for this study were collected as part of a longitudinal study of Syrian and Iraqi refugees who were recruited within 1 month of their arrival to the United States 13 14 15 16 17 and are continuing to be followed for up to 5 years postresettlement. 18 Data for the present study were collected 1 to 2 years ( *M* = 1.7 years) postresettlement. Data were collected in person, at participants' homes. Inclusion criteria for the present study included (1) willing and able to provide informed written consent in either Arabic or English; (2) adults ages 18 to 65 years; (3) originally from Syria and having been admitted through a refugee program to the United States, and (4) able to understand Arabic and/or English. Table 1 provides a summary of the demographic characteristics of the study sample ( *n* = 53). All procedures described herein were approved by the Institutional Review Board (IRB) at Wayne State University (IRB#012416B3F).

**Table 1:** Characteristics of study population

Variable	Total sample ( *n* = 53)
Female	27 (50.9%)
Male	26 (49.1%)
Marital status	
Married, *n* (%)	40 (75.5%)
Single, *n* (%)	3 (24.5%)
Employment	
Employed, *n* (%)	20 (37.7%)
Unemployed, *n* (%)	32 (60.4 %)
Health (self-reported)	
Excellent, *n* (%)	10 (18.9%)
Very good, *n* (%)	12 (22.6%)
Good, *n* (%)	17 (32.1%)
Fair, *n* (%)	11 (20.8%)
Poor, *n* (%)	3 (5.7%)
City origin	
Aleppo, *n* (%)	3 (5.7%)
Baghdad, *n* (%) a	1 (1.9%)
Damascus, *n* (%)	7 (13.2%)
Daraa, *n* (%)	24 (45.3%)
Hama, *n* (%)	2 (3.8%)
Homs, *n* (%)	12 (22.6%)
Idlib, *n* (%)	2 (3.8%)
English speak	
Not at all, *n* (%)	13 (24.5%)
Not well, *n* (%)	22 (41.5%)
Well, *n* (%)	18 (34%)
Very well, *n* (%)	0
English Write	
Not at all, *n* (%)	14 (26.4%)
Not well, *n* (%)	17 (32.1%)
Well, *n* (%)	22 (41.5%)
Very Well, *n* (%)	0

a This participant was originally from Baghdad, however, was living in Syria prior to the beginning of the 2011 Syrian crisis.

### Procedure

Data were collected via scheduled home visits by research staff. All measures were self-report and were completed in either validated English or Arabic (both languages were present on the same form). A bilingual clinician research assistant was present during the entire duration of data collection at refugees' homes.

### Measures

*Demographics Questionnaire:* A demographics questionnaire was included to assess age, gender, religious affiliation, employment, marital status, the highest level of education, occupation, substance use, medical conditions, medication use, subjective ratings of overall health, subjective rating of ability to speak and write English, and city of origin within Syria.

*Traumatic Events:* The Life Events Checklist for the Diagnostic and Statistical Manual of Mental Disorders, Fifth Edition (DSM-5; LEC-5) screens for 16 potential traumatic events in the respondent's lifetime. 19 It also includes an additional item screening for any other extremely stressful event not indicated by the other 16 items. There is no formal scoring method or total score for the LEC-5; rather, it identifies if the respondent has directly experienced, witnessed, learned about, or been exposed via occupation to the events listed. In the present study, only items that were endorsed 1 = “Happened to me” or 2 = “Witnessed it” were included in the data analysis. For each participant, a total score was computed of the number of traumatic events endorsed as either directly experienced or witnessed.

*Posttraumatic Stress:* The Posttraumatic Stress Disorder Checklist (PCL-5) is the most used measure to assess posttraumatic stress based on DSM-5 criteria. Recent studies on the psychometric properties of the PCL-5 cite a cutoff score of 33 considered to be indicative of clinically significant symptomology. 20 Cronbach's α for this sample was 0.92.

*Current Life Stressors* : The Postmigration Living Difficulties Questionnaire (LDQ) assesses the current life stressors of refugees and asylum seekers. 21 Items include communication, discrimination, access to medical and social services, isolation, and safety. There is no formal scoring method or total cut-off score for the LDQ. For the purpose of this study, a cumulative score was used to assess for greater living difficulties experienced since migrating to the United States. Cronbach's α was found to be 0.92 for this sample.

*Sleep Quality:* Overall sleep quality is measured using the Pittsburgh Sleep Quality Index (PSQI) which is an instrument for psychiatric practice and research. 22 This scale consists of 19 self-rated items which assess seven components of sleep quality within the past month including subjective sleep quality, sleep latency, sleep duration, sleep efficiency, sleep disturbance, use of sleep medication, and daytime dysfunction. A PSQI global score of 5 or more indicates clinically significant sleep disturbances. 22

*Disrupted Nocturnal Behaviors (DNB):* The Pittsburgh Sleep Quality Index Addendum (PSQI-A) was used to assess the frequency of DNB and has been shown to have good internal consistency and convergent validity. 23 PSQI-A assesses seven specific DNB symptoms including frequency of hot flashes; general nervousness; memories or nightmares of traumatic experience; severe anxiety or panic not related to traumatic memories; bad dreams not related to traumatic memories; episodes of terror or screaming during sleep without fully awakening that are considered hallmark symptoms of PTSD; and total scores ≥ 4 are considered symptomatic PTSD. 24

### Data Analysis

Basic descriptive statistics were generated for the main variables of PSQI, PSQI-A, PCL-5, LEC-5, and LDQ ( Table 2 ). Prior to any analyses data were checked for normalcy, outliers, and distribution. Results showed that individual scores missing more than two responses were excluded from analysis and for individual scores missing two or less responses mean-replacement was utilized. Correlational analyses were conducted between PSQI, PSQI-A, and the variables of PTSD symptom severity, postmigration life difficulties, and previous trauma experienced. A stepwise linear regression analysis was conducted to examine the role of overall sleep quality, PTSD-specific sleep disturbances, current living difficulties, and the number of preimmigration traumatic events directly experienced or witnessed due to the presence of overall PTSD symptomology.

**Table 2:** Summary of measures used for study population

Variable	Total Sample ( *n* = 53)
PSQI	
(Mean ± SD)	5.15 ± 2.49
PSQI ≥ 5 ( *n* , %)	(33, 63%)
PSQI-A	
(Mean ± SD)	6.67 ± 6.67
PSQI-A ≥ 4 ( *n* , %)	(31, 58%)
Living difficulties questionnaire (LDQ)	
(Mean ± SD)	20.06 ± 15.63
Stressful events experienced (LEC-5)	
(Mean ± SD)	2.73 ± 2.50
PCL-5	
(Mean ± SD)	32.16 ± 10.99
PCL-5 > 33 ( *n* , %)	(24, 49%)

Abbreviations: LDQ, Postmigration Living Difficulties Questionnaire; LEC-5, The Life Events Checklist for DSM-5; PCL-5, Posttraumatic Stress Disorder Checklist- 5; PSQI, Pittsburgh Sleep Quality Index; PSQI-A, Pittsburgh Sleep Quality Index-Addendum.

PSQI *n* = 52, PSQI-A *n* = 46 LDQ *n* = 52, LEC5 *n* = 41, PCL-5 *n* = 49.

## Results

Average posttraumatic stress scores on the PCL-5 ( *M* = 32.16, SD = 10.99) approached the clinical cutoff (33) for PTSD diagnosis. The average PSQI score ( *M* = 5.15, SD = 2.49) surpassed the clinical cutoff of 5 indicating significant sleep disturbances. 23 Descriptive statistics for all measures are denoted in Table 1 .

As can be seen in Table 3 , Pearson correlation indicated that the PSQI-A was significantly positively correlated with PSQI, postmigration living difficulties, and posttraumatic stress ( *p* < 0.01). Posttraumatic stress was also positively correlated with postmigration living difficulties and PSQI ( *p* < 0.01). Trauma exposure was significantly positively correlated with postmigration living difficulties ( *p* < 0.05).

**Table 3:** Correlations among overall sleep quality, PTSD-related sleep disturbances, current living difficulties, trauma experienced, and overall presence of PTSD symptomology

	PCL-5	PSQI-A	LDQ	PSQI	Total stressful events experienced
1. PCL-5	–	0.65 b	0.56 b	0.42 b	0.18
2. PSQI-A		–	0.45 b	0.59 b	0.11
3. LDQ			–	0.27	0.37 a
4. PSQI				–	0.05
5. LEC-5					–

Abbreviations: LDQ, Postmigration Living Difficulties Questionnaire; LEC-5, The Life Events Checklist for DSM-5; PCL-5, Posttraumatic Stress Disorder Checklist- 5; PSQI, Pittsburgh Sleep Quality Index; PSQI-A, Pittsburgh Sleep Quality Index-Addendum.

Note: PCL-5 *n* = 49, PSQI-A *n* = 46, LDQ *n* = 52, PSQI *n* = 52, LEC-5 *n* = 41.

a Correlation is significant at the *p* < 0.05 (2-tailed).

b Correlation is significant at the *p* <0.01 (2-tailed).

Of the four variables examined as possible predictors, in the stepwise regression, two variables were selected in a step-wise fashion for entry into the prediction of PTSD symptom severity (see Table 4 ). LDQ was entered in the first step of the regression model and it accounted for 39% of the variance in the model ( *F* [1,31] = 20.14, *p* < 0.01). LDQ significantly predicted current PTSD symptomology (B = 0.44, *t* [30] = 4.49, *p* < 0.01). PSQI-A scores were added in the second step of the model, and 51% of the variance of overall PTSD symptoms was attributed to LDQ and PSQI-A scores ( *F* [1, 30] = 15.79, *p* < 0.01). The PSQI-A produced a significant increment in the prediction of current PTSD symptoms over LDQ alone, with an *R* ^2^ change value of 0.12 *(p* < 0.01) as shown in Table 4 . The PSQI and LEC-5 did not make a significant contribution to the prediction of PTSD symptom severity.

**Table 4:** A stepwise linear multiple regression analysis predicting current PTSD

	Variable	B	SE B	β	t	*R* ^2^	F	∆ *R* ^2^	F
Step 1						0.39 ^a^			20.14
	Constant	23.31	2.49		9.37				
	LDQ	0.44	0.10	0.63	4.49				
Step 2						0.51 ^a^	15.79	0.12 ^a^	7.34
	Constant	21.79	2.34		9.34				
	LDQ	0.30	0.10	0.42	2.85				
	PSQI-A	0.66	0.25	0.40	2.71				

Abbreviations: LDQ, Postmigration Living Difficulties Questionnaire; LEC-5, The Life Events Checklist for DSM-5; PSQI, Pittsburgh Sleep Quality Index; PSQI-A, Pittsburgh Sleep Quality Index-Addendum.

Note: ^a^ *p* < 0.001, Step 1 *df* (1, 31); Step 2 *df* (1, 30), *n* = 33.

Excluded variables: LEC-5, PSQI-Total.

## Discussion

The study's main findings are that there was an association between DNB and overall sleep quality and that even 1 year after migration Syrian refugees experience significant sleep disturbances and symptoms of PTSD. These findings corroborate previous studies in male military veterans which reported a strong correlation between PTSD-related sleep disturbances, as measured by the PSQI-A, and overall poor sleep quality, as measured by the PSQI. 25

Previous studies found sleep disturbances to be an important part of PTSD symptomatology particularly recurrent nightmares and trouble sleeping among refugees. 26 27 Our study corroborates these findings, and in addition, we found that the refugees' sleep disturbances are more influenced by their current daily life struggles such as communication difficulties, discrimination, and worry about economic security, and not necessarily by more distal prior traumas they experienced. This novel finding is significant, as it suggests that there is a relationship between current life stressors and PTSD-related sleep disturbances. Hence, the daily living struggles of refugees may be more detrimental to refugees' sleep than the traumatic events experienced they experienced in the past.

Participants who reported higher symptoms of PTSD-related sleep disturbances also reported higher posttraumatic stress, similar to what has been observed in refugee and asylum seekers in Melbourne, Australia, where the severity of sleep disturbances was positively correlated with more severe posttraumatic stress. 28 Furthermore, a recent study on refugees from Syria and Iraq who were settled in Australia also demonstrated that there was a high prevalence of sleep disturbances related to pre- and postimmigration trauma. 29 These Australian studies, however, only assessed sleep by a stand-alone item that defined sleep disturbance as difficulty falling or staying asleep only—it did not use the validated PSQI-A measure as was done in this present study. Thus, the present study is more sensitive to the relationship between specific sleep disturbances and the presence of PTSD symptom severity in refugees and represents a significant addition to the literature.

As expected, PTSD-related sleep disturbances were found to be strongly correlated with the severity of PTSD symptomology among this refugee group, and the presence of DNB was a strong predictor of the presence of PTSD symptoms. Moreover, when the PSQI-A was added to the stepwise multiple regression model, it significantly enhanced the prediction of PTSD symptom severity by current stressors alone. This suggests that sleep disturbances significantly impact PTSD symptom severity, even beyond the level that would be predicted from the current level of stress. The implication of this finding underscores the importance of sleep for well-being, specifically for refugees who have PTSD. While causation cannot be determined based on current findings, these results suggest that this population of refugees' sleep and PTSD symptomology are highly interrelated.

### Implications

Sleep disturbances may be a clinical indicator of PTSD severity and may signal a need for integrative care models that address both sleep disturbances and PTSD symptoms such as cognitive behavioral therapy (CBT) and cognitive processing therapy (CPT) as a treatment for PTSD. 30 31 While it would be ideal to offer treatment for PTSD, continuing medical, psychiatric, or psychological services are not always readily available to refugee populations. 32 However, integrative care models and interprofessional teams trained to provide treatment for PTSD including CBT and CPT will likely be helpful in improving refugees' mental health and reducing disability. 30 Many of the refugees and other traumatized populations might only have access to primary care services or feel more comfortable sharing their symptoms including sleep disturbances with their primary care physician. Also, basic psychoeducation on sleep hygiene may help alleviate some symptoms until more comprehensive care is available. In a study of adult working women with sleep problems, it was found that sleep hygiene education led to improved sleep quality outcomes. 33

Refugees continue to face challenges in their host countries including worry regarding economic stability, social isolation, changes in their family functioning and structure, limited and difficult access to education, cultural barriers, 34 and experienced hostility and racism. 35 36 Our findings indicate that these stressors are likely impacting the severity of PTSD and associated sleep disturbances. Policies should focus on reducing barriers to education, work opportunities, and access to health care. Providing assistance to refugees to address these living difficulties may improve the resettlement experience as well as overall health, specifically for those with elevated PTSD symptoms.

The findings of this study can be utilized in both clinical and research settings. Currently, research on Syrian refugees living in the United States and refugees in general is minimal. Continued research is critical, as refugees often have their own unique stressors and challenges compared with other traumatized populations. Understanding the many psychological, physical, and social hardships Syrian refugees face will allow for better outcomes in resettlement, and perhaps encourage governments and agencies to address the many social issues that refugees face. Future research can also address to what extent, addressing sleep impairments can improve PTSD symptoms and the level of functioning among refugees and other traumatized populations.

## Limitations

The greatest limitation of our study was the relatively small sample size. There were difficulties in contacting and arranging home appointments with refugees 1-year postinitial migration. Furthermore, the U.S. immigration policy of 2017 significantly limited the number of Syrian refugees entering the country. Additionally, the population in this study had a unique situation of living in an area with a large Arabic-speaking community. Thus, their experiences will likely differ compared with Syrian refugees in other parts of the country where language and communication would be more of a barrier. In addition, this study did not compare the sample to the general population who are nonrefugees.

Limitations in the measures themselves may have also been a factor in the study. The LEC-5 may not fully capture the nature and severity of traumatic experiences. Merely counting events does not account for the absolute level of trauma experienced, how frequently these individual events were experienced, or the overall impact on the refugee. Also, the cross-sectional nature of the study is a substantial limitation of the study. This data are only a snapshot of the refugees' symptomology. There are no previous data to have a baseline of refugees' sleep and PTSD symptomology prior to arriving in the United States. All measures were self-reports, with no objective measure of symptomology. Furthermore, the findings cannot determine causation, but rather only the association between the measures.

## Strengths

This study examined the relationship between sleep disturbances, postmigration living difficulties, and previous trauma experienced with PTSD symptom severity in Syrian refugees in the United States. Our study had the advantage of a homogenous group of participants of young age, both sexes, coming from a similar background, with relatively similar traumatic and postmigration stressors, and the same timing of leaving Syria and arriving in the host country. The study adds to the knowledge of overall sleep quality and DNB disturbances and their likely impact on refugees.

## Conclusion

In conclusion, it was found that sleep disturbances, especially DNB were strongly associated with PTSD symptomology and current life stressors among Syrian refugees living in the United States. We also found that DNB were a significant predictor of the presence of PTSD symptoms in this population. These findings provide important insight into the underlying struggles refugees may face in their host countries and highlight key challenges that can be targeted by future therapeutic studies.
